# Protective effect of hydroxysafflor yellow A against acute kidney injury via the TLR4/NF-κB signaling pathway

**DOI:** 10.1038/s41598-018-27217-3

**Published:** 2018-06-15

**Authors:** Juan Bai, Jinyi Zhao, Dongxiao Cui, Fan Wang, Ying Song, Lianghua Cheng, Kai Gao, Jin Wang, Long Li, Shujun Li, Yanyan Jia, Aidong Wen

**Affiliations:** 1 Xijing Hospital, Medical University of the Air Force, Department of Pharmacy, Xi’an, Shaanxi 710032 China; 2 Shaanxi University of Chinese Medicine, Department of Pharmacy, Xianyang, shaanxi 712046 China; 3 Xijing Hospital, Medical University of the Air Force, State Key Laboratory of Cancer Biology, Department of Gastrointestinal Surgery, Xi’an, Shaanxi 710032 China

## Abstract

This study aimed to evaluate the protective effect of hydroxysafflor yellow A (HSYA) on ischemia/reperfusion (I/R)-induced acute kidney injury via the TLR4/NF-κB pathway, both *in vitro* and *in vivo*. Rats were subjected to removal of the right kidney and I/R injury to the left kidney. Rats subjected to renal I/R injury were treated with HSYA at 0.5 h prior to I/R injury. Renal function, histopathological analysis, and cells apoptosis were measured *in vivo. In vitro*, proximal renal tubular cells (HK-2) were subjected to hypoxia/reoxygenation (H/R). Apoptotic cell death and inflammatory cytokines, Toll-like receptor 4 (TLR4), and nuclear factor (NF)-κB expression were determined. Treatment of I/R rats with HSYA markedly reduced the levels of serum creatinine and blood urea nitrogen, attenuated renal cell apoptosis, alleviated changes in renal tissue morphology, and reduced IL-1β, TNF-α, and caspase-3 release. *In vitro*, HSYA effectively decreased NF-κB p65 and inflammatory cytokines, such as IL-1β, TNF-α, and IL-6. Thus, HSYA can protect renal function from I/R injury by ameliorating acute kidney injury and partly by promoting tubular cell survival via the TLR4/NF-κB pathway. These results suggest that HSYA can be used to prevent I/R-induced acute kidney injury.

## Introduction

Acute kidney injury (AKI) is a clinical syndrome with very high mortality rates. AKI-associated costs account for 5% of hospital expenditures^1^ and 1% of overall health expenditures^2^ in developed countries. Renal ischemia/reperfusion (I/R) injury is a common cause of acute kidney injury. The pathophysiology of I/R injury in the kidney is very complex, involving free radicals, inflammatory media, calcium overload, apoptosis, and energy metabolism dysfunction^3,4^. The prognosis for patients with I/R injury is poor and there is no effective therapy for counteracting this injury. Traditional Chinese medicines exhibit many biological activities, including antioxidant, anti-apoptotic, anti-inflammatory, anti-microbial, anti-viral, and anti-cancer effects. Therefore, traditional Chinese medicine may be useful for rapid and effective drug discovery to protect against I/R injury.

The yellow pigment of *Carthamus tinctorius* L. is predominantly used for activating blood and dissolving stasis. HSYA is a water-soluble monomer that can be extracted from *C. tinctorius* L. and is a type of chalcone glycoside (molecular formula, C_27_H_32_O_16_; molecular weight, 612.53 g/mol)^5^. HSYA is the main chemical component of *C. tinctorius* L., which has long been used to prevent and treat cardiovascular disease in traditional Chinese medicine^6^. It was demonstrated to have broad physiological and pharmacological functions, including anti-inflammation and apoptosis inhibition effects^7–9^. Additionally, studies showed that HSYA played an important role in protecting organs. Therefore, we investigated the effect of HSYA on renal I/R injury in rats and further explored its mechanism.

Inflammation has been recognized as one of the most critical pathophysiological responses involved in renal I/R injury. Toll-like receptor 4 (TLR4) is a key regulator of the pro-inflammatory transcription factor (NF-κB) and plays a dominant role in mediating sterile kidney damage following renal I/R injury^10^. Renal tubular epithelial cells and vascular endothelial cells are key cell types in the biological action of TLR4. High TLR4 expression is observed in kidneys subjected to I/R. NF-κB upregulation may be caused by infiltrating macrophages and intrinsic renal cells, and is important in mediating sterile kidney damage following renal I/R injury^10,11^. TLR4 is crucial in initiating the influx of various immune cells such as polymorphonuclear leukocytes, lymphocytes, dendritic cells, and macrophages, into the damaged interstitium (pro-inflammatory phase)^12,13^. NF-κB is a member of the family of pleiotropic transcription factors which play important roles in regulating the expression of genes involved in various cell processes, including inflammation^14,15^. NF-κB activation is considered a hallmark of acute inflammatory processes^15^. TLR4 activation induces the expression of NF-κB-dependent proinflammatory cytokines such as tumor necrosis factor-α (TNF-α), interleukin-6 (IL-6), and IL-1β^16^. These cytokines induce tubular epithelial cell necrosis and renal tubular atrophy^17^. TLR4 activation further triggers NF-κB transcription, which downregulates inflammatory genes, upregulates anti-inflammatory genes, and induces leukocyte apoptosis^18^. IκB kinase (IKK) activation leads to the dissociation of NF-κB from IκB and its subsequent activation. Therefore, suppressing abnormal immune responses through the TLR4/NF-κB pathway may be an effective measure for attenuating renal I/R injury.

This study aimed to evaluate the ability of HSYA to mitigate kidney dysfunction induced by I/R injury. Our results showed that HSYA prevented renal functional alterations induced by I/R injury partly by inhibiting activation of the TLR4/NF-κB pathway.

## Results

### HSYA improves renal function and histopathology after I/R injury

**Figure 1:**
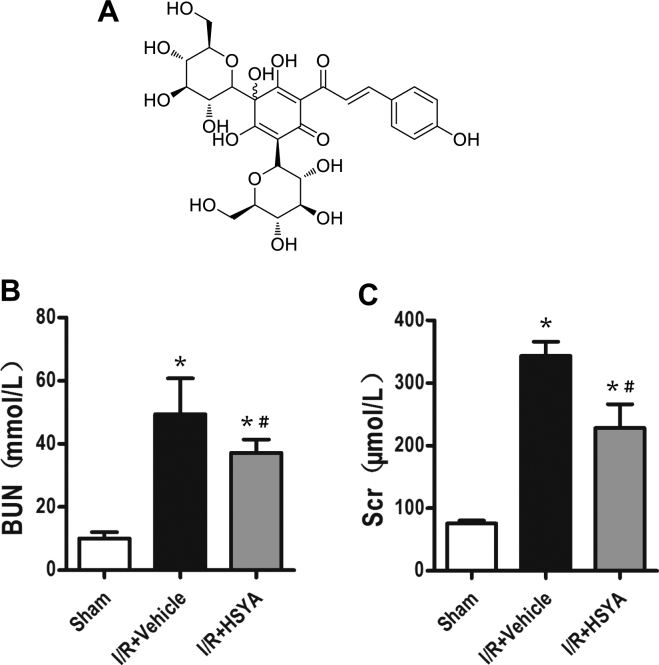
Effect of HSYA on kidney function tests in I/R-induced AKI in rats. (**A**) Chemical structure of hydroxysafflor yellow A. (**B**,**C**) Increases in blood urea nitrogen (BUN) and serum creatinine (Scr) levels at 24 h after I/R were abrogated by HSYA (92 mg/kg, administered intraperitoneally) (n = 8 rats/group). *P < 0.05 vs. sham-operated (Sham) group; ^#^P < 0.05 vs. I/R + Vehicle group.

Based on the results of serum assays, the levels of blood urea nitrogen (BUN) and serum creatinine (SCr) were significantly increased in the I/R + Vehicle group. Compared to the sham group, at 24 h after renal reperfusion, the I/R + Vehicle group developed drastic renal dysfunction as indicated by increased BUN (56.38 ± 3.16 mmol/L) and Scr (359.25 ± 57.66 µmol/L) levels. Rats pre-treated with HSYA (I/R + HSYA group) did not exhibit significant increases in BUN (38.31 ± 2.78 mmol/L) and SCr (255.00 ± 18.76 µmol/L) levels. Thus, the renal function changes induced by I/R were significantly ameliorated by treatment with HSYA (Fig. 1B,C).

**Figure 2:**
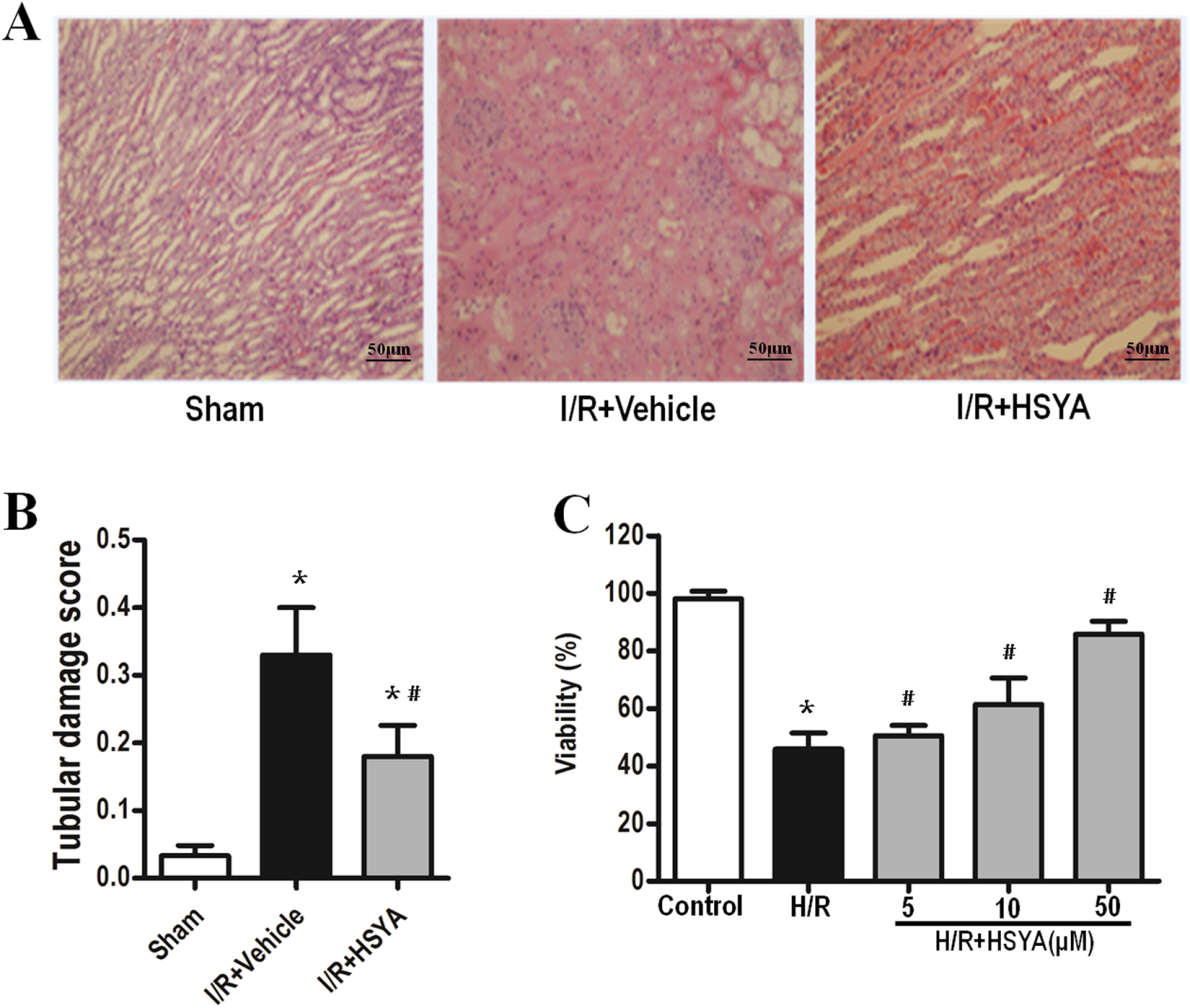
Effect of HSYA on kidney injury after I/R administration. (**A**) Representative photomicrographs (hematoxylin and eosin staining; magnification, 200×) of renal sections from rats under various experimental conditions. (**B**) Semi-quantitative assessment of tubular injury. All data are presented as the means ± SD (n = 6); *P < 0.05 versus Sham; ^#^P < 0.05 versus I/R + Vehicle. (**C**) The effect of HSYA on HK-2 cell viability was tested by MTT assay. Cells in the control group were considered 100% viable. Data are presented as the means ± SD (n = 3); *P < 0.01 vs. Control group; ^#^P < 0.01 vs. H/R group.

Hematoxylin and eosin (H&E) staining results revealed kidney tubular necrosis, dilation, and cast formation in the I/R + Vehicle group. Treatment with HSYA at 0.5 h prior to I/R injury reduced the severity of tubular injury (Fig. 2A). Quantitative analysis showed that rats pre-treated with HSYA exhibited a markedly lower renal tubular injury score (Fig. 2B).

### HSYA reduces apoptosis after I/R injury

**Figure 3:**
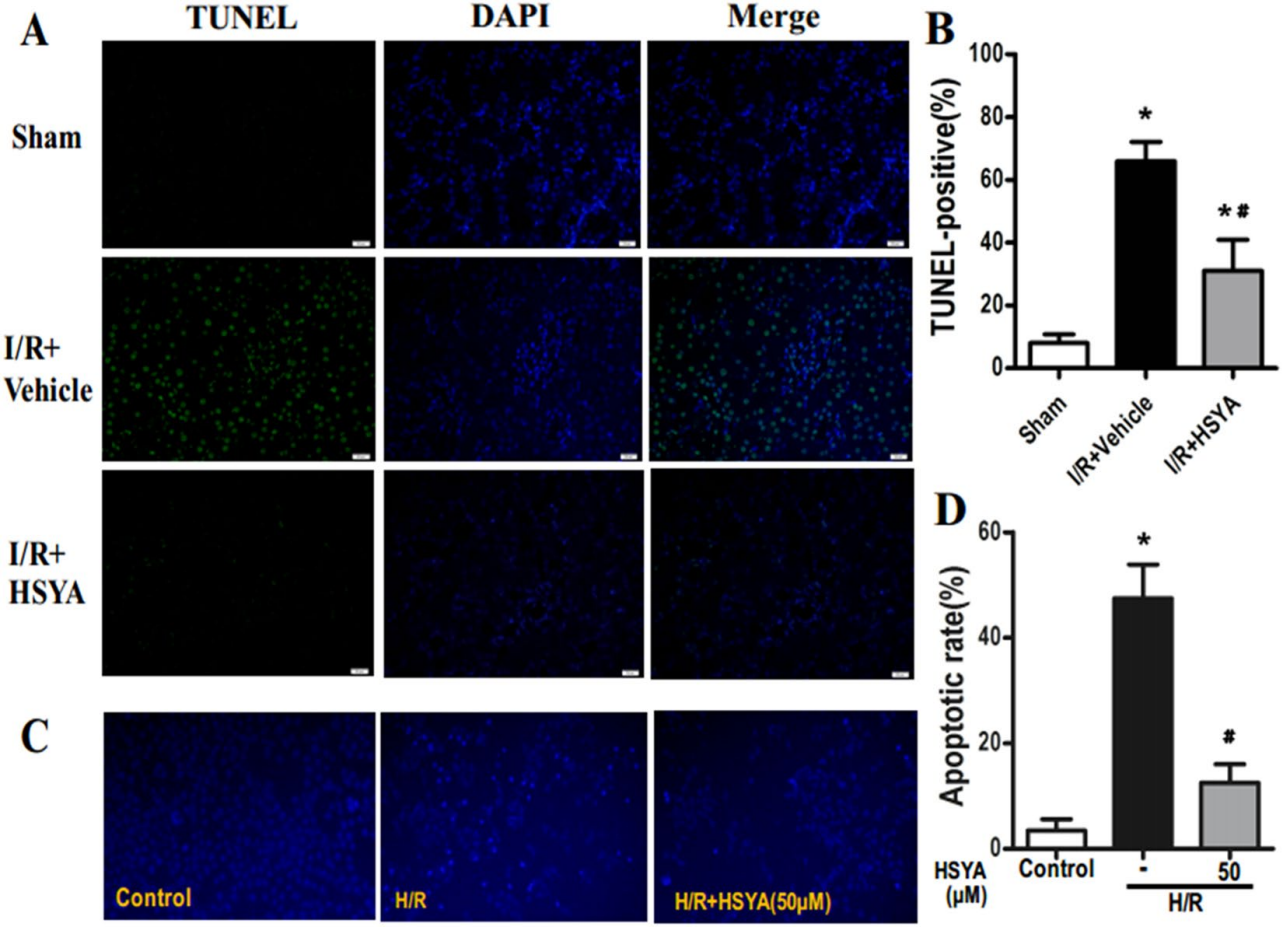
HSYA preconditioning ameliorates tubular apoptosis induced by I/R. (**A**) Representative images (magnification, x400) of renal TUNEL assay from rats subjected to sham group, I/R + Vehicle group, and I/R + HSYA. (**B**) The percentage of TUNEL-positive cells in kidney sections increased following I/R and decreased following treatment with HSYA (*P < 0.05 vs. Sham; ^#^P < 0.05 vs. I/R + Vehicle. n = 8 rats per group). (**C**) Anti-apoptotic effects on HK-2 cell following H/R injury were confirmed by Hoechst 33258 staining. Quantitative analysis of apoptosis is shown in (**D**). Data were presented as the means ± SD and were representative of three independent experiments; *P < 0.01 versus Control; ^#^P < 0.01 versus H/R.

*In vivo*, TUNEL staining of renal sections was performed to visualize DNA fragmentation *in situ*. In the I/R + Vehicle group, the TUNEL assay showed a remarkable increase in TUNEL-positive cells in I/R-injured kidneys, whereas pre-treatment with HSYA reduced the number of TUNEL-positive cells (Fig. 3A). In rats pre-treated with HSYA, the percentage of TUNEL-positive cells in renal sections decreased from 69.40 ± 11.74% to 26.25 ± 0.52% (Fig. 3B).

*In vitro*, to test whether HSYA exerts a protective effect against H/R-induced apoptosis, we evaluated the apoptosis status of HK-2 cells. Hoechst 33258 staining showed that the proportion of apoptotic cells was significantly up-regulated by H/R treatment, whereas it was markedly reduced by HSYA (Fig. 3C,D). In summary, HSYA effectively inhibits H/R-induced renal proximal tubular cell apoptosis.

The proliferation of HK-2 cells was also evaluated by using the CCK-8 assay. HSYA pretreatment showed no effect on HK-2 cell viability under normal conditions. In contrast, H/R markedly inhibited HK-2 proliferation, which was significantly enhanced by HSYA in a dose-dependent manner (Fig. 2C). These results revealed that HSYA was not cytotoxic against HK-2 cells and enhanced the cell survival reduced by H/R treatment.

### HSYA inhibits cytokine expression

**Figure 4:**
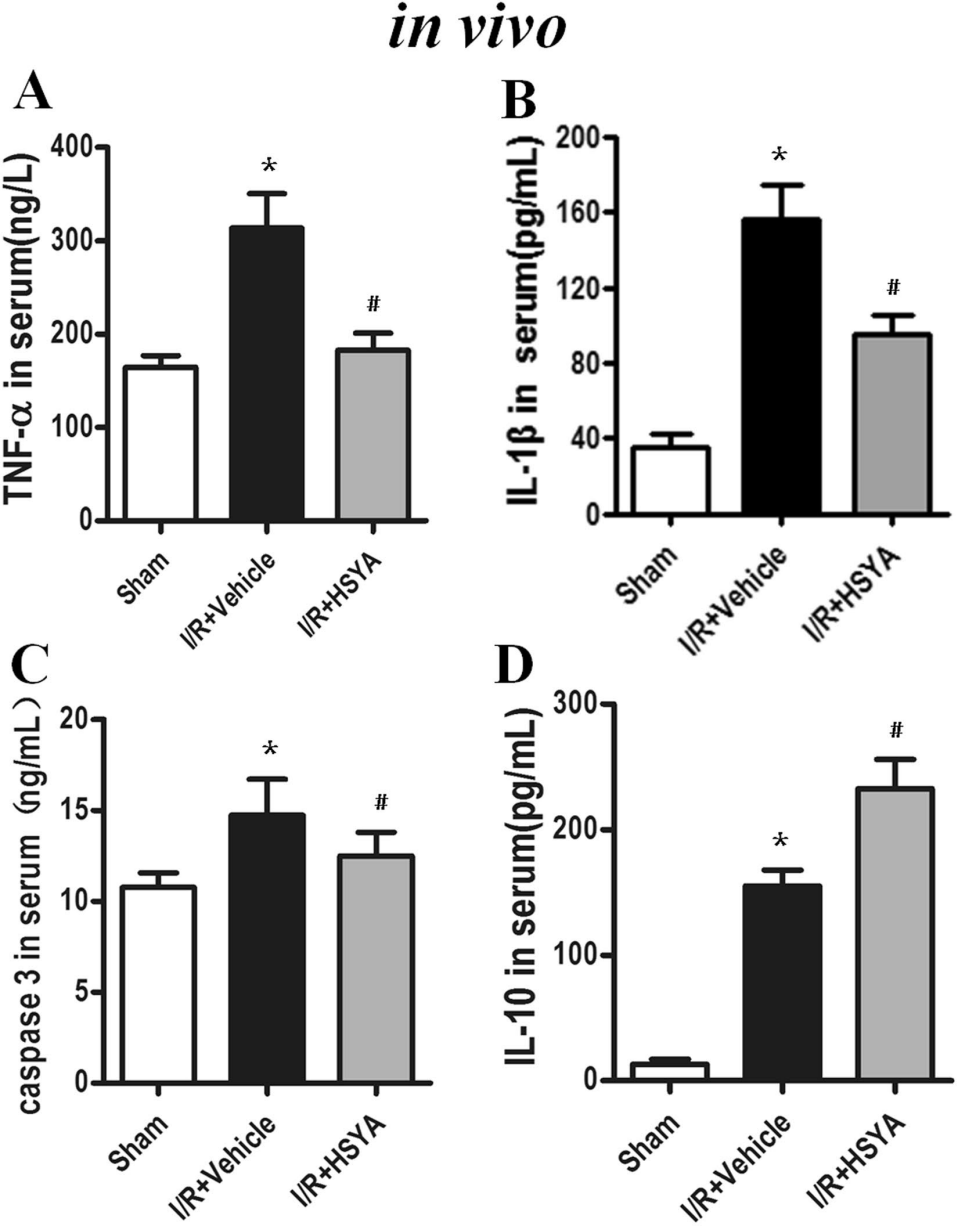
Effect of HSYA on TNF-α (**A**), IL-1β (**B**), caspase-3 (**C**), and IL-10 (**D**) levels after I/R challenge. Quantitation of TNF-α, IL-1β, caspase-3, and IL-10 in renal tissue was performed by ELISA. Data are represented as the mean ± SD of 8 animals in each group and experiments were repeated three times. *P < 0.05 vs. sham group; ^#^P < 0.05 vs. I/R + Vehicle group.

**Figure 5:**
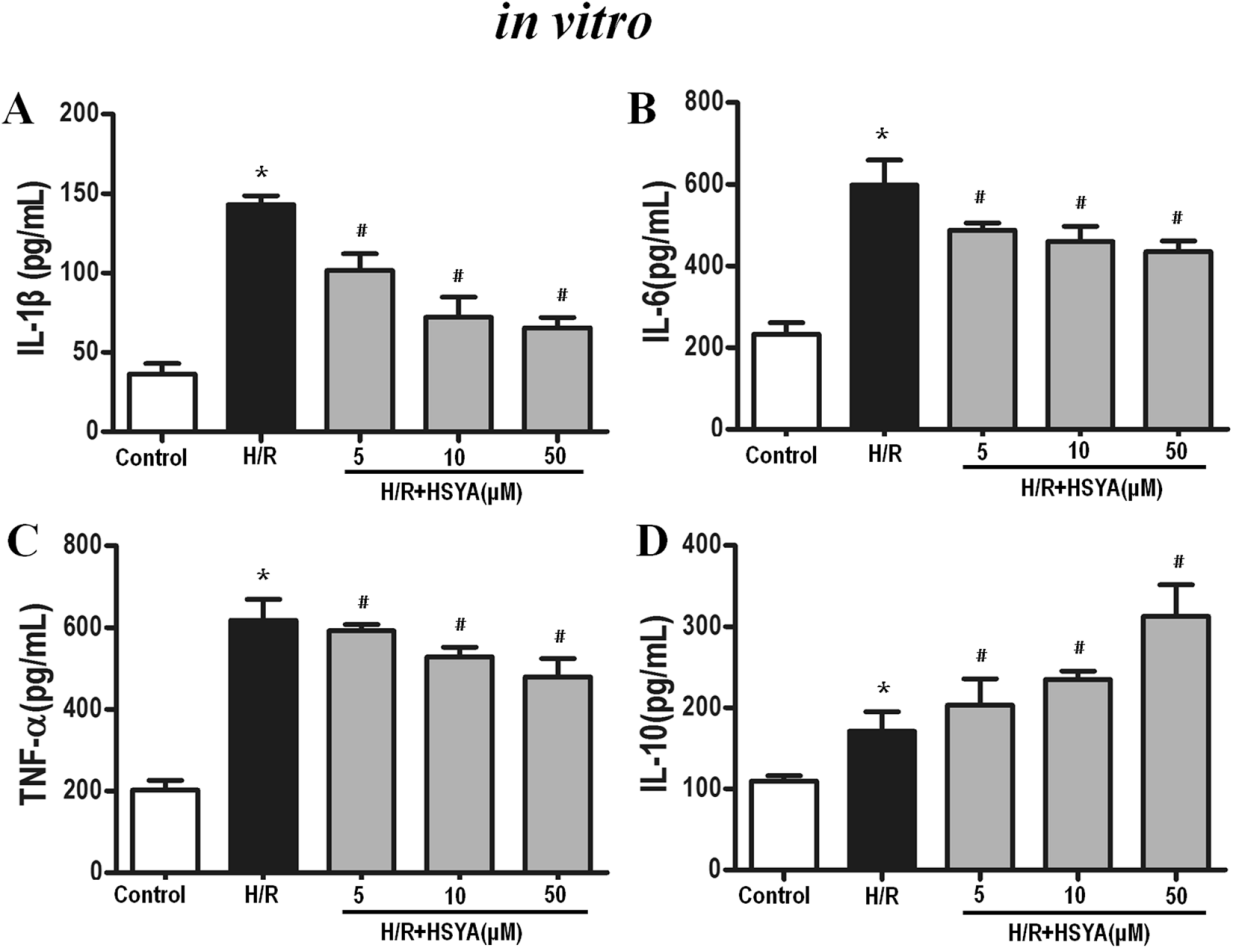
Effect of HSYA on IL-1β (**A**), IL-6 (**B**), TNF-α (**C**), and IL-10 (**D**) release induced by H/R in HK-2 cells. Cells were treated with H/R with or without HSYA (5, 10, and 50 μM) for 24 h. A volume of 100 μL of culture medium in each group was removed to measure the levels of IL-1β, IL-6, TNF-α, and IL-10 using ELISA kits. Data are presented as the means ± SD (n = 3); *P < 0.01 vs. Control group; ^#^P < 0.01 vs. H/R group.

To further estimate the HSYA-mediated inhibition of TLR4 inflammasome activation, we analyzed the levels of inflammatory cytokines induced by I/R *in vivo* and H/R *in vitro*. Compared to the sham group, the I/R group showed drastically higher levels of TNF-α, IL-1*β*, and caspase-3 in the rat kidneys (*P* < 0.01). However, treatment with HSYA significantly inhibited I/R-induced TNF-α, IL-1*β*, and caspase-3 production (shown in Fig. 4A–C), but increased IL-10 release (Fig. 4D). As shown in Fig. 5A–C, HSYA treatment at concentrations of 5–50 *μ*M notably suppressed the release of IL-1*β*, IL-6, and TNF-α in H/R-stimulated HK-2 cells in a dose-dependent manner (*P* < 0.05). Moreover, Fig. 5D shows that, as expected, HSYA treatment at concentrations of 5–50 *μ*M markedly increased the secretion of IL-10 in the supernatant (*P* < 0.05).

### HSYA suppresses the inflammatory response by inhibiting the activation of TLR4/NF-κB

**Figure 6:**
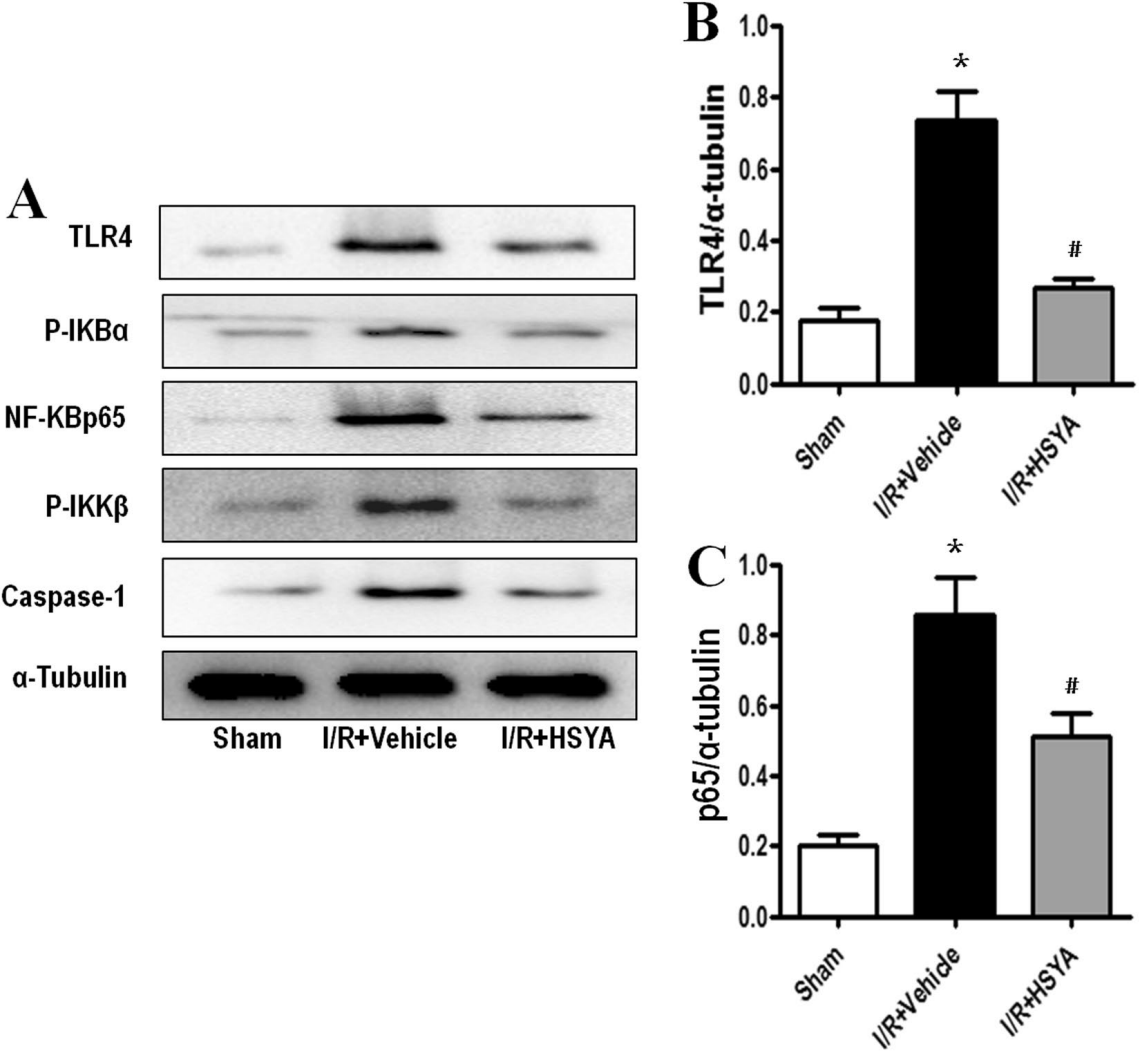
Effects of HSYA on activation of the TLR4/ NF-κB signaling pathway in I/R-induced AKI. Representative western blots showing the effects of HSYA on TLR4 and NF-κB expression in the kidney after 45 min of ischemia followed by 24 h of reperfusion. α-Tubulin was used to show equal amounts of protein loading in each lane. (**A**) Representative western blots showing the effects of HSYA on TLR4, NF-κBp65, phosphorylation of IKKβ, IκBα, and caspase-1 expression. Relative band densities of (**B**) TLR4 and (**C**) NF-κBp65 to the mean value of the control. *P < 0.05 versus Sham; ^#^P < 0.05 versus I/R + Vehicle.

**Figure 7:**
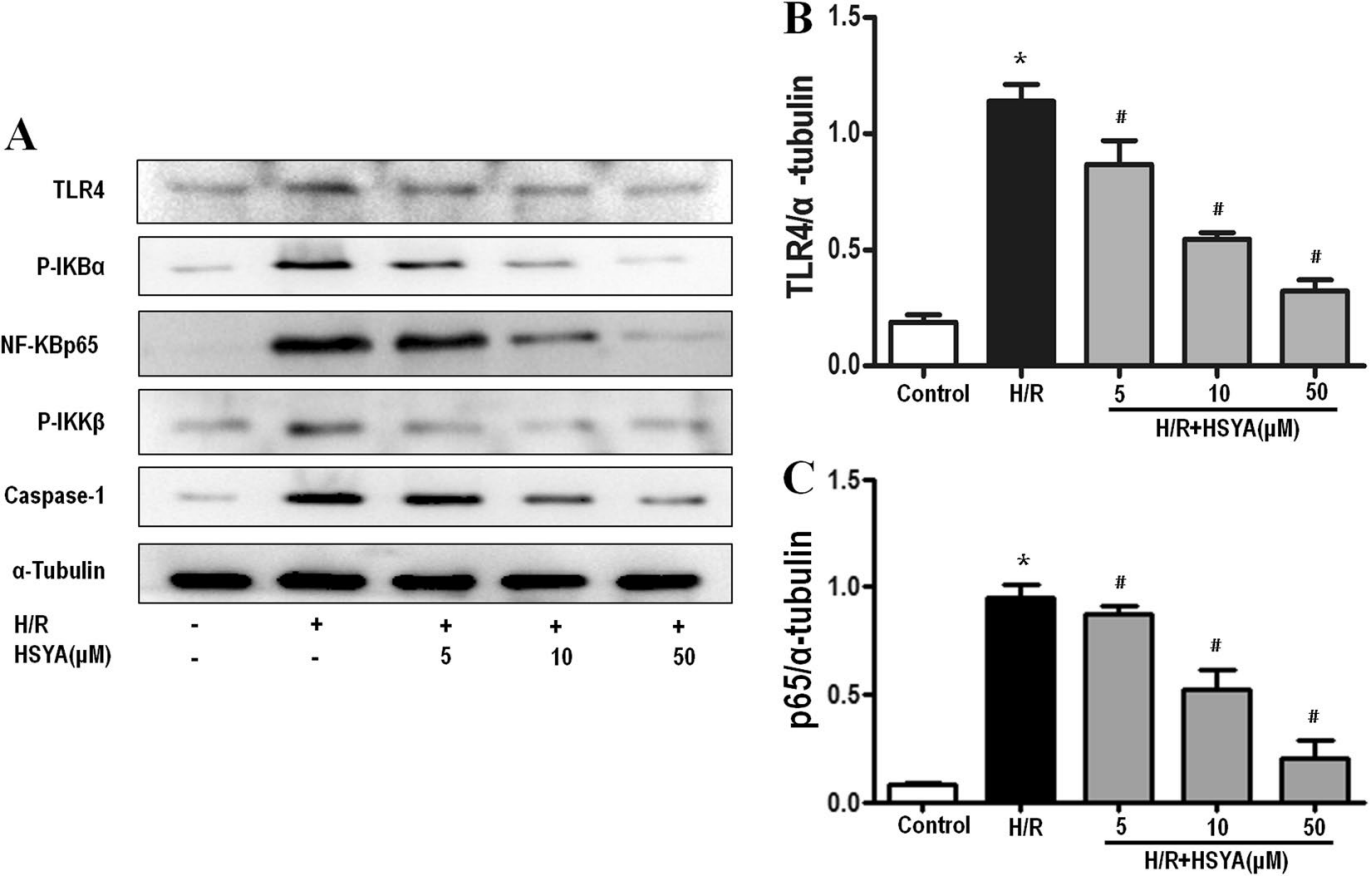
Effect of HSYA on TLR4/NF-κB signal pathway in H/R-induced HK-2 cells. HK-2 were incubated in the presence or absence of different concentrations of HSYA (5, 10, 50 mM) for 24 h, and then incubated with or without H/R for another 24 h. α-Tubulin was used to show equal amounts of protein loading in each lane. The expression levels of TLR4, NF-κBp65, phosphorylation of IKKβ, IκBα, and caspase-1 (**A**) in cell lysates were determined by western blotting. Relative band densities of (**B**) TLR4 and (**C**) NF-κBp65 to the mean value of the control. All data represent the means ± SD from three separate experiments. *P < 0.01 vs. Control group, ^#^P < 0.01 vs. H/R group.

The TLR4/NF-κB signaling pathway plays a critical role in inflammatory cytokine production. In the TLR4-mediated pathway, activated TLR4 increases phosphorylation of the downstream kinase, p-IKKβ, which then induces IκBα phosphorylation, resulting in nuclear translocation of NF-κBp65 and regulation of immune/inflammatory responses. To investigate the mechanism by which HSYA inhibits I/R-induced production of inflammatory cytokines, we assessed the expression of TLR4 and related proteins in the NF-κB pathway *in vitro*. As shown in Figs 6 and 7, the protein expression of phospho-IKKβ, phospho-IκBα, and NF-κBp65 was increased in the I/R-induced group compared to in the control group, indicating that NF-κBp65 activity was increased. Administration of HSYA down-regulated the expression of NF-κB p65, phosphorylated IKKβ, and IκBα. These results suggest that HSYA has a protective effect on rats and HK-2 cells regarding I/R-induced AKI by suppressing the NF-κB signaling pathway.

## Discussion

The occurrence of AKI continues to increase, resulting in higher rates of mortality and morbidity because of the lack of effective therapies^19–21^. In recent years, accumulating evidence has suggested that renal I/R injury is the most common cause of AKI and that plants used in traditional Chinese medicine and compounds isolated from these medicinal plants possess potent anti-inflammatory properties^22,23^.

HSYA is the major active component of *C. tinctorius* L., which has been widely used to treat ischemia diseases for thousands of years in China. Previous studies demonstrated that HSYA protected against kidney injury, which may be related to its anti-inflammatory action^24,25^. Unfortunately, scientific evidence supporting the use of HSYA is inadequate and the underlying mechanism remains unclear. In this study, we evaluated how HSYA protects rats against I/R renal injury.

Our study showed that pre-administered HSYA alleviated renal injury in I/R by decreasing BUN and SCr levels and attenuating renal cell apoptosis. TNF-α levels were decreased when HSYA was utilized for I/R injury in rats. As a pro-inflammatory factor, TNF-α is closely related to extensive tubular damage^26,27^. HSYA decreases TNF-α expression to enable survival after I/R renal injury. Additionally, HSYA increased the level of the anti-inflammatory cytokine IL-10 in a dose-dependent manner *in vitro*. Thus, HSYA-mediated protection against renal I/R injury is related to inflammatory reactions.

TLR4 signaling plays a critical role in renal I/R injury and results in NF-κB activation^28,29^. TLR4 is a receptor for pathogen-associated molecular patterns and damage-associated molecular patterns expressed on the cell surface and plays an important role in activating the inflammatory reaction. HSYA inhibits the expression of TLR4 in HK-2 cells undergoing H/R injury. These results suggest that HSYA confers protection from H/R injury through TLR4.

NF-κB is a transcription factor activated by TLR4^30^ and plays a key role in regulating inflammation^31^. NF-κB induces the production of pro-inflammatory factors, such as IL-1β and TNF-α. HSYA significantly suppressed the expression of NF-κBp65, phosphorylated IKKβ, and IκBα induced by I/R injury in a dose-dependent manner. Furthermore, the pro-inflammatory factors IL-1β, IL-6, and TNF-α were decreased following exposure to HSYA. In contrast, the anti-inflammatory factor IL-10 was increased, suggesting that NF-κB participates in protecting the kidneys from I/R injury.

Overall, HSYA can protect the kidneys against I/R injury and has the potential to be developed as a drug for preventing renal I/R injury. The TLR4/NF-κB pathway is involved in HSYA-mediated renal protection.

In summary, our results revealed that administration of HSYA had a protective effect against renal I/R injury, which may be ascribed to blockade of the TLR4/NF-κB pathway resulting in attenuation of the inflammatory response. Our results suggest that HSYA is a candidate for treating renal I/R injury, and thus may be an appropriate therapy for I/R disease. However, whether prophylactic and therapeutic administration of HSYA can effectively prevent AKI incidence and improve clinical outcomes in patients requires further analysis.

## Materials and Methods

### Chemicals and reagents

Hydroxysafflor yellow A (purity = 96.5%, HPLC) was purchased from the Chinese Academy of Food and Drug Control (Beijing, China). MTT Assay kits was procured from Shaanxi MYBiotech Co. Ltd. TechnoMart (Shanghai, China). 4′,6-Diamidino-2-phenylindole (DAPI) (9542) was obtained from Sigma Chemical Co. (St. Louis, MO, USA). The terminal deoxynucleotide transferase dUTP nick end labeling (TUNEL) assay kit (ApoBrdu DNA fragmentation assay kit) (K403-50) was procured from Biovision, Inc. (Milpitas, CA, USA). TNF-α, IL-1β, IL-6, IL-10, and caspase-3 ELISA Kits were purchased from R&D Systems (Minneapolis, MN, USA). Antibodies against TLR4, phospho-IκBα, NF-κB p65, phospho-IKKβ, caspase-1 and α-tubulin were obtained from Abcam (Cambridge, UK). BUN and SCr assay kit reagents were purchased from the Institute of Jiancheng Bioengineering (Nanjing, China). Dulbecco’s modified Eagle’s medium (DMEM) and other cell culture supplies were purchased from Gibco (Grand Island, NY, USA). All materials for sodium dodecyl sulfate-polyacrylamide gel electrophoresis (SDS-PAGE) were obtained from Bio-Rad Laboratories, Inc. (Hercules, CA, USA). The hypoxic/ischemic chamber was obtained from Billups-Rothenberg, Inc. (San Diego, CA, USA). All other chemicals used were of analytical grade and purchased from Sigma Chemical Co.

### Animals

Adult Sprague-Dawley rats (purchased from the Department of Laboratory Animal Science, Medical University of the Air Force, Xi’an, China) weighing 250–300 g were used in the present study. All experimental rats were kept in an environmentally controlled breeding room for 5 days before being used in the experiments and were fed with standard laboratory food and water.

### Ethics statement

The experimental procedures involving animals in this study were approved by the Animal Ethics Committee at the Medical University of the Air Force. Experimental protocols were followed with strict adherence to the regulations set forth by the National Institutes of Health guidelines for the use of laboratory animals (NIH publication no. 85–23, National Academy Press, Washington, DC, USA, revised 1996).

## Experimental design

### *In vivo* study

#### Rat model of renal I/R injury

Pathogen-free, adult male Sprague-Dawley rats were fasted overnight. The rat model of renal I/R injury and surgical procedures involved were similar to previously described methods^32–35^. All rats were anesthetized with sodium chloral hydrate (85 mg/kg intraperitoneally) (Rhone Merieux Limited, Essex, UK) and placed in a prone position on a warming pad at 37 °C to perform the surgical procedures. Sham-operated rats (sham group) were only subjected to removal of the right kidney, whereas rats in the I/R group and I/R + HSYA group were also subjected to acute I/R injury to the left kidney induced by clamping the renal artery with non-traumatic vascular clamps for 45 min. Reperfusion was established by removing the clamp. Blood was collected from the eye socket at 24 h after reperfusion and the left kidney was removed at 24 h. Rats were sacrificed by decapitation and exsanguination at 24 h after the I/R procedure. The kidneys were harvested for further analysis.

The rats were randomly and equally divided into 3 groups as follows: Sham-operated group (n = 8), the right kidney was extirpated for sham-operated animals, but neither clamping nor infusion was performed in the left kidney; I/R + Vehicle group (n = 8), rats subjected to renal I/R injury were treated with saline as the Vehicle; I/R + HSYA group (n = 8), rats subjected to renal I/R injury were treated with HSYA (92 mg/kg, intravenous administration) at 0.5 h prior to I/R injury.

#### Histopathological examination

Transverse slices of the left kidneys were fixed in 10% buffered formalin and embedded with paraffin and sectioned at 4 µm thickness. The sections were deparaffinized and hydrated gradually, and then stained with hematoxylin-eosin (H&E). At least 5 images were randomly captured at 200× magnification using Leica Application Suite software (ver.3.4.1, Leica Microsystems GmbH, Wetzlar, Germany). One hundred intersections were examined for each kidney, and a score of 0–3 was given for each tubular profile. The scoring method has been described previously^36^ and was as follows: 0, normal histology; 1, tubular cell swelling, brush border loss, nuclear condensation, with up to one-third of the tubular profile showing nuclear loss; 2, same as score 1 but greater than one-third and less than two-thirds of the tubular profile showing nuclear loss; and 3, greater than two-thirds of the tubular profile showing nuclear loss. The total score for each kidney was calculated by adding all 100 scores, with a maximum score of 300.

#### Biochemical measurements

Renal function injury and inflammatory cytokines were assessed in rats subjected to I/R injury. BUN and SCr, as important indices of renal injury severity, were used to assess renal function. Blood samples were obtained by retro-orbital puncture at 24 h following reperfusion. Serum was separated by centrifugation at 2700 × *g* and at 4 °C, and SCr and BUN levels were determined by the Clinical Laboratory at Xijng Hospital. Blood samples were stored at −80 °C for further analysis.

The levels of the inflammatory mediators TNF-α, IL-1β, caspase-3, and IL-10 in the serum were quantified using a specific ELISA kit for rats according to the manufacturer’s instructions.

#### Determination of apoptosis

Renal cell apoptosis was detected by terminal deoxynucleotidyl transferase-mediated dUTP nick-end labeling (TUNEL) assay using tissue paraffin blocks. Renal slides were incubated with the TUNEL reaction mixture in a humidified chamber for 60 min at 37 °C in the dark. Renal sections were then rinsed three times in phosphate-buffered saline (PBS), and the nuclei were stained with DAPI at a concentration of 300 nM. Apoptosis was quantified by calculating the percentage of TUNEL-positive nuclei from among the total nuclei in an average of 20 high-power fields for each section in a blinded manner.

#### Western blot analysis

The kidney tissue samples were homogenized on ice followed by centrifugation at 12,000 × *g* for 30 s. The proteins were extracted according to the instructions of the total protein extraction kit. Protein concentrations were determined by using the BCA protein assay kit. Equal amounts of protein were separated by SDS-PAGE and analyzed by western blotting using specific antibodies to TLR4, NF-κBp65, caspase-1 and phosphorylated IκBα and IKKβ. Horseradish peroxidase-conjugated goat anti-rabbit IgG antibody (Merck, Kenilworth, NJ, USA) was used as a secondary antibody. After washing the membranes four times with Tris-buffered saline containing Tween 20, the blots were visualized with electrochemiluminescence-plus reagent and α-tubulin was used as a loading control. Immunoblots were visualized and recorded with a Bio-Rad detection system, and gray density analysis was completed with the Image J program (NIH, Bethesda, MD, USA).

### *In vitro* study

#### Cell culture

Human renal proximal tubular epithelial cells (HK-2 cells) were obtained from the European Collection of Cell Cultures (Salisbury, UK) and cultured at 37 °C in an atmosphere of 5% CO_2_ and 95% O_2_ in DMEM containing 10% fetal bovine serum and 1.0% penicillin-streptomycin solution. When they reached confluence in 3–4 days, the cells were trypsinized (0.25% trypsin-EDTA) and used for further experiments.

#### Construction of the HK-2 cell injury model

To model ischemia-like conditions *in vitro*, HK-2 cells were exposed to ischemia by replacing the medium with an ‘ischemic buffer’ (5 mM HEPES, 137 mM NaCl, 4 mM KCl, 1 mM MgCl_2_, and 1.5 mM CaCl_2_, pH 7.0). The cells were then incubated in a hypoxic/ischemic chamber at 37 °C for 60 min in a humidified atmosphere of 5% CO_2_ and 95% N_2_. Finally, the cells were incubated in culture medium in an incubator with 95% air and 5% CO_2_ for an additional 24 h as described previously^37–40^. For all experiments, the cells were plated at an appropriate density according to the experimental design and grown for 24 h before treatment. The cells were divided into 5 groups as below: (1) Control group: cells were treated with PBS; (2) Hypoxia/reoxygenation (H/R) group: cells were treated as described above to mimic ischemia-like conditions; (3) HSYA + H/R group (5, 10, 50 μM): cells were treated with different concentrations of HSYA for 24 h and then subjected to H/R conditions.

#### MTT assay for cell viability

Cell viability was determined using a cell counting kit-8 (CCK-8) assay according to the manufacturer’s instructions (MYBiotech). HK-2 cells (5 × 10^3^ cells/well) were plated into 96-well plates and pretreated with HSYA (5, 10, and 50 μM) for 24 h prior to exposure to H/R exposure, after which 10 μL CCK-8 was added and the cells were incubated for 4 h. Absorbance was measured at 450 nm using a microplate reader (Bio-Rad Laboratories, Inc.).

#### Determination of apoptosis

For the Hoechst 33258 staining assay, HK-2 cells were washed with ice-cold PBS, fixed in 10% neutral-buffered formalin for 10 min at 37 °C, and washed again with ice-cold PBS. HK-2 cells were then exposed to Hoechst 33258 (2 µg/mL in PBS) and incubated for 20 min at room temperature. The stained cells were washed three times with PBS and examined under a fluorescence microscope with an appropriate filter. Images were captured at a magnification of ×200 using an Olympus microscope (1 × 71; Olympus, Tokyo, Japan).

#### Cytokine assays *in vitro*

HK-2 cells were seeded in 6-well culture plates at a density of 5 × 10^4^ cells/mL and pretreated with various concentrations (5, 10, 50 μM) of HSYA for 24 h, followed by H/R. The levels of TNF-α, IL-1β, IL-6, and IL-10 were quantified using a commercial ELISA kit according to the manufacturer’s instructions. All cytokines were measured separately in supernatants collected after H/R stimulation.

#### Western blot analysis

Cells incubated and treated in 6-well plates were lysed in 150 μL RIPA lysis buffers containing protease inhibitor (PMSF, 1 mM) and phosphatase inhibitor (TIANDZ 80809-1, 1%) on an ice-bath for 30 min. The lysate was centrifuged at 10,000 × *g* for 10 min at 4 °C, the supernatant was collected and proteins were quantified using the BCA protein assay kit (Beyotime Biotechnology, Nanjing, China). After the proteins were transferred to a polyvinylidene difluoride membrane, the membranes were blocked with 5% nonfat dry milk and incubated overnight with the appropriate primary antibodies (anti-TLR4, anti-p-IKKβ, anti-p-IKBα, anti-NF-κBp65, and anti-caspase-1 antibodies), followed by washing four times with Tris-buffered saline containing Tween 20. The membranes were then incubated with secondary horseradish peroxidase-conjugated goat anti-rabbit IgG. This was followed by four washes with Tris-buffered saline containing Tween 20. The blots were visualized with electrochemiluminescence-plus reagent and α-tubulin was used as a loading control.

### Statistical analysis

Data are expressed as the mean ± standard deviation (SD). Numerical data are presented as the mean ± SD from at least three individual experiments. All statistical analyses were performed using SPSS 16.0 software (SPSS, Inc., Chicago, IL, USA). Comparisons between two groups were performed using an independent-sample *t*-test. Experiments with more than two groups were compared by analysis of variance followed by Tukey’s post-hoc test. P values < 0.05 were considered statistically significant.
